# Learn to Live Again: A Pilot Study to Support Women Experiencing Domestic Violence

**DOI:** 10.3390/ijerph22050714

**Published:** 2025-05-01

**Authors:** Jacqui Cameron, Delia Rambaldini-Gooding, Kirsty Vezinias, Brooke Smith, Maria Corsiglia, Sarah Beale

**Affiliations:** 1 School of Social Sciences, University of Wollongong, Wollongong, NSW 2500, Australia; deliarg@uow.edu.au (D.R.-G.);; 2 Department of Social Work, University of Melbourne, Parkville, VIC 3010, Australia; 3 Barnardos, Warrawong, NSW 2502, Australia; mcorsiglia@barnardos.org.au (M.C.); sbeale@barnardos.org.au (S.B.)

**Keywords:** domestic violence, women, children, therapeutic program, mental health, L2LA, pilot study

## Abstract

Purpose: The prevalence of domestic violence is increasing, and it is becoming more common for women who have experienced domestic violence to access support programs in their community. Learn to Live Again (L2LA) is an eight-week therapeutic program facilitated by Barnardos, which is provided through community support services in NSW, Australia. The program is designed for women who have experienced or continue to experience the traumatic effects of family and domestic violence. Methods: The pilot study involved collecting survey data from former participants and semi-structured interviews with current participants and facilitators. Data collection occurred between June and August 2023. Data collection included both face-to-face and online versions of the program. Results: All participants reported positive experiences of L2LA. The main benefits of the program for participants included connecting with women in similar situations, learning skills and strategies to cope with their experiences, sharing their lived experiences of domestic violence, and reconnecting with their children. Facilitators also had positive experiences of facilitating the program and observing the positive changes women experienced. Although, challenges were also identified and included managing the group dynamics, small group sizes, and managing trauma experiences in a group setting. Conclusion: The results indicate participants as well as facilitators had positive experiences of L2LA. Participants valued the program and felt that it helped them to begin the healing process and improve their overall wellbeing. Women were very passionate about recommending the program to other women. Facilitators observed many positive changes in the women throughout the program. However, L2LA challenges included the small group size, managing the dynamics of the group, and the range of trauma within the group as well as children being present. A larger evaluation of this program is required to confirm these findings.

## 1. Introduction

Domestic and family violence (DFV) is one of the most pervasive forms of violence worldwide [1]. In Australia, approximately 27 percent of women have experienced violence by an intimate partner since the age of 15 [2]. DFV impacts women’s physical and mental health [3]. Domestic and family violence refers to any behaviour in an intimate or family relationship that is violent, threatening, coercive, or controlling causing a person to live in fear. It can include but is not limited to physical, sexual, emotional, economic, or technology-facilitated abuse [4]. In Australia, DFV is the leading cause of emergency room presentations and hospital admissions among women aged 15–44 [5]. Survivors/victims of DFV frequently report poor mental health including symptoms of PTSD, anxiety, depression, self-harm, and suicidal thoughts [6,7,8]. Women often experience disruption from informal social support networks such as family and friends [3] as well as financial issues due to economic abuse and/or inability to maintain employment [9,10]. The accumulation of these psychological, social, and emotional traumas perpetrated by intimate partners often prevents women from disclosing DFV and seeking support [11]. In addition, women fear repercussions from partners and the removal of their children by authorities, further compounding social isolation and feelings of self-blame [11,12]. As a result, DFV can also impact women’s parenting and attachment with their children thereby compounding trauma [13,14]. Exposure to DFV can affect children’s physical and mental wellbeing, cognitive development, behaviour, and schooling and can lead to homelessness [11,15,16]. These children are also more likely to experience other forms of child abuse including sexual abuse [16]. The cumulative effects of DFV and other forms of child abuse can result in long-term psychosocial issues such as depression, anxiety, social isolation, difficulty forming relationships, and exposure to violence [17].

Interventions supporting victims/survivors post identification to address this complex trauma caused by DFV are paramount to their recovery and healing [18]. Effective interventions can address short- and medium-term physical, emotional, and social impacts on victims/survivors and their children [19,20] and can prevent long-term harm [21]. Disclosing to health and other practitioners [22], then engaging in group intervention programs, gives women an opportunity to share lived experiences and connect with women who have similar experiences, thereby addressing social isolation and rebuilding women’s self-esteem [23,24]. Creating a safe and supportive environment can also enhance the mothers’ relationship with their children by developing coping and communication tools [25] and improving their overall health and wellbeing [15].

Online group interventions for victims/survivors of DFV emerged in response to COVID-19 and have remained a feature of social services due to their ability to bring interventions to scale and offer services to women in rural or remote communities [26,27]. The efficacy of online interventions in contexts such as mental health is clearer [28,29,30]; however, the literature focused on the efficacy of online interventions for survivors/victims of DFV is limited. For victims/survivors experiencing DFV, there are concerns around online safety, privacy, and trust in the service and other participants [27,31].

The literature on group interventions delivered in both online and face-to-face modes is limited. Nor do studies explore whether there is flexibility in interventions to accommodate group dynamics or women’s changing needs and circumstances. Much of the literature also focuses on individual health and wellbeing outcomes for women and does not take into consideration how women’s engagement may affect relationships with their children. Using the NIH [32] definition of a pilot study, this pilot study evaluated the Learn to Live Again (L2LA) program delivered by Barnardos Australia, a group-based intervention that supports victims/survivors of DFV.

L2LA is delivered as a small group intervention both online via teleconferencing and face-to-face.

The purpose of the L2LA program is to reconnect women to themselves, their bodies, their families, and to their community. By healing these disrupted connections, the program aims to strengthen the bond between women and their children and begin to heal their experiences of trauma. The groups are intentionally small with a maximum of 4–6 women designed to create a safe and nurturing environment to support trauma-informed practice. The pilot study of L2LA provides an evidence base for future expansion of the program. Thus, the aim of the pilot study was to answer the following two questions: What is the experience of women participating in the L2LA program? What is the experience of facilitating the L2LA program?

## 2. Materials and Methods

### 2.1. Study Design

A pilot program often involves systematically collecting and analysing data about the program’s activities, characteristics, and outcomes [33]. This process can help determine the program’s effectiveness, identify areas for improvement, and inform decisions about whether to scale up the initiative. Key aspects of this pilot study include:

1. Data Collection: Gathering quantitative and qualitative data from various sources, such as surveys, interviews, and performance metrics.
2. Analysis: Assessing the data to understand how well the pilot program met its objectives and identifying any challenges or successes.
3. Feedback: Using the insights gained to make informed decisions about the program’s future, including potential adjustments or broader implementation.

This pilot study focused on item #3 feedback to support future potential adjustments and broader implementation.

### 2.2. Study Recruitment

The study recruitment included the following:

Former participants: Former program participants were recruited through Barnardos via an online survey. Barnardos sent the survey link to protect participants’ privacy. The survey, conducted using Qualtrics [34] included eleven open- and closed-ended questions. Participants received two reminder emails to complete the survey.

Current participants: All women in the face-to-face program (running at the time of the study) at Barnardos were invited to participate in the study. Facilitators explained the project and arranged meetings to obtain consent and schedule interviews. Participants received an information sheet and had access to Barnardos staff and the research team if they had further questions. The interview schedule was brief and included eleven questions.

Facilitators of the program: Current facilitators were recruited directly from Barnardos. They received an information and consent form and were invited to participate in interviews either online or face-to-face. The interview schedule was brief and included eleven questions.

### 2.3. Study Data Collection

**Figure 1:**
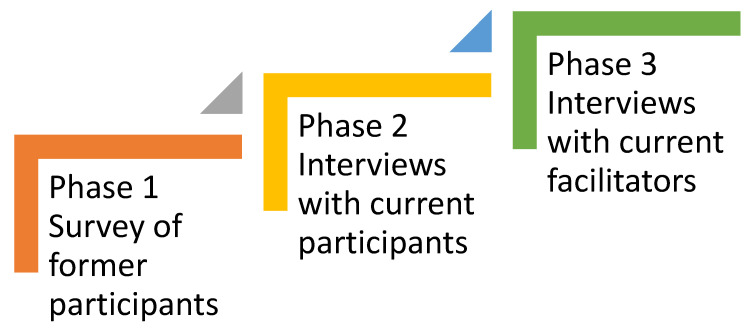
Pilot study phase.

The data collection included three phases (Figure 1).

The quantitative data were collected through the use of surveys, involving questions on the experience of the program and program satisfaction. The qualitative interviews conducted with participants and facilitators provided an opportunity for a more in-depth understanding of the experience of the online L2LA program from the perspective of women and facilitators. Interviews ranged from 15 to 20 min. The survey took on average 10 min to complete.

The quantitative survey data targeted former participants of the program, the interviews targeted current participants of the program, as well as current facilitators of the program.

### 2.4. Study Analysis

The pilot study included participants from both versions of the program, i.e., face-to-face and online. The pilot study used a combination of data analysis including simple descriptive statistics (frequency/percentages) [4] to analyse the survey data in Qualtrics [34] and thematic analysis [35] to analyse the qualitative data once the interviews were fully transcribed and saved as password-protected transcripts. Utilising NVivo 12 (2022) [36], inductive thematic analysis was conducted following Braun and Clarke’s [35] six phases of analysis to identify themes presented in the results section.

### 2.5. Study Ethics

The pilot study was approved by the UOW Human Research Ethics Committee (HREC ID 2023 006).

### 2.6. Study Intervention

*“Please persevere and keep going, this group will change your entire mindset and allow you to be able to live again, to be happy and feel safe, because you deserve to be free, and you are worthy of happiness”.*

Barnardos Australia’s Learn to Live Again (L2LA) program is an eight-week therapeutic group designed for women with children who have or continue to experience the traumatic effects of family and domestic violence and abuse. The purpose of the group is to reconnect women to themselves, their bodies, their families, and to the community around them. By healing these disrupted connections, the group aims to heal and strengthen the bond between women and their children providing a platform for women to begin to support their own children’s experiences of lived trauma. The program is underpinned by trauma and attachment theory, grief and loss, and ambiguous loss, as well as utilizing the concepts of Dr. Peter Levine’s [37] Somatic Experiencing and Dr. Allan Wade’s [38] Responses and Resistance. More recently, the principles of David Mandel’s Safe & Together model [39] of informed domestic violence practice have been incorporated throughout the program. 

The draft L2LA program was developed in 2016, where we sought information from services and their clients on what they needed and to avoid duplication or a disjointed service experience. The Learn to Live Again pilot group was initially delivered on the NSW South Coast in 2017 to a cohort of 6 women with existing case management support from Barnardos South Coast. The pilot group provided consultative feedback on what worked and what they would like to see included in the group, as well as co-designing the naming and logo for the group, with the decision being how they felt the 8 weeks helped them to “Learn to Live Again”, and the logo representing the group of women holding hands in support of each other. More than one hundred and fifty women have attended Learn to Live Again groups throughout NSW and ACT since 2017. Forty-four practitioners have completed a 2-day Train the Facilitator program since 2018 across multiple Barnardos Children’s Family Centre sites enhancing the capacity to offer the support of Learn to Live Again with fidelity, potential, and hope. The table below (Table 1) outlines the program topics covered in the L2LA program.

## 3. Results

The table below (Table 2) presents an overview of the participants who participated in data collection (*n* = 38). This included a brief satisfaction survey of former participants (*n* = 24), followed by one-to-one interviews with current participants (*n* = 8) and interviews with facilitators (*n* = 6).

### 3.1. Survey Results

In total, *n* = 24 former participants opened the survey link; however, not all women answered every question, so the total number of responses for each question varies between questions. A summary list of the questions from the survey is provided in Table 3.

The first question of the survey asked women what motivated them to complete the program, women provided the following open-text responses:

- *“I felt supported in a safe environment”;*
- *“The more knowledge I had I felt stronger in myself”;*
- *“Learning new things”;*
- *“The opportunity to recover and regain my self worth”;*
- *“It had a lot of helpful information for learning to live again”.*

Two women also provided more detailed responses below.

*“I was going through a horrible separation which saw myself and my children live in refuges as my children’s father sold our family home leaving us homeless. I was referred to Barnardos by one of the caseworkers I was working with as they believed I would benefit from the program.”*

*“I needed to continue my life after leaving my husband and be able to talk to other women from domestic violence situations. I needed to be happy again.”*

A summary of satisfaction results is provided below.

- *n* = 24 women found the program beneficial.
- *n* = 18 women reported the program delivery structure including group size, group duration, topics, and activities were appropriate.
- *n* = 13 women rated their overall experience as very good or excellent.
- *n* = 11 women reported a strong level of engagement in the program and rated their engagement as ‘very’ engaged.
- *n* = 7 reported the program met their expectations.

The final survey question explores the topic areas used within the program were ranked by women as most/least helpful included: “My Kids and I—Giving and receiving Love”; “Patchwork Life—Goals and reality”; “Pathways of change—love myself”, and “The Healing Tree—Strength Through Growth”.

### 3.2. Interview Results

#### L2LA Participants

Women in the online (*n* = 2) and face-to-face (*n* = 6) programs participated in interviews and described their experiences of L2LA. Three main themes were identified including (1) *“Get some light on everything”*, which was related to making connections with other women, the provision of a supportive environment, and facilitators’ care and compassion; (2) *“Came together as a perfect puzzle”* focused on practical program components, self-care, and the accessibility of the program; and (3) *“Moving on and moving forward”*, related to improvement in women’s wellbeing and the desire to recommend the program to other women. Women identified similar experiences across the online and face-to-face versions of the program. The notable difference was women in the online program felt that the length of each session was too short. Please see Table 4, participant themes.

### 3.3. “Get Some Light on Everything”

#### 3.3.1. Making Connections

Participants discussed either “*connecting*” (Participant 3) *or “bonding”* (Participant 5) with women in similar situations as the most important aspect of the program. As explained by one participant.

*“It is a really special group because we were around people who have experienced similar things, and it definitely made you feel better and more confident in sharing some of the things you had been through”*

(Participant 6).

Another participant stated,

*“The support is the biggest word like the sense of community and support that were in it together and that we are here as a team, it was a team environment it wasn’t like I was just a customer taking what I wanted, like I felt like I was there to contribute just as much as I’d like to receive.”*

(Participant 2—Online)

Sharing lived experiences of domestic violence not only helped women to establish connections between each other but helped women to reconnect with themselves and validate their experiences.

*“to hear some of the stuff that they had been through, even though I had gone through almost the same, for some things in particular, hearing it come from somebody else, gives you a different kind of empathy to reflect on yourself.”*

(Participant 1—Online)

#### 3.3.2. Providing a Safe and Nurturing Environment

Participants reflected on the importance of a safe and nurturing environment to heal from domestic violence, and this was identified as a positive aspect of *L2LA*. A participant stated below:

*“It was nice to come into such a positive and nurturing environment where everyone was so connected, it was something I looked forward to coming to”*

(Participant 6).

The nurturing environment helped participants realise that they deserve respect and began to appreciate themselves.

*“I feel like it was a part of learning to know that I could deserve such a nice thing”*

(Participant 5).

Participants reflected that the program helped them to change their mindset and value themselves.

*“…it really helps when you’re stuck in a mindset, and someone will say something or bring it out in a different way and change the way you think about it”*

(Participant 3).

The supportive environment provided participants with a sense of validation for their feelings and actions.

*“…I felt really validated as well that I could share stuff that had happened, and they could stand up and be like yeah that’s shit, that’s not how life is meant to be and how I feel is similar”*

(Participant 2 Online).

Having the opportunity to listen, reflect, and communicate with other women who have experienced domestic and/or family violence supported women to begin the healing process.

#### 3.3.3. Facilitators’ Connection with Women

Participants valued the facilitators’ engagement, approach, and skills. Facilitators’ connection with women enhanced their experience of the program. Participants appreciated the gentle and unrushed approach from the facilitators. In the words of one participant:

*“I never got rushed or hurried when I was having particularly bad weeks when I just wanted to talk about stuff. They always had time for me and others to offload whatever we had to talk about during that week. It was always enough time to hear us out and listen”*

(Participant 2).

Participants described the facilitators as having the skills to create a relaxed, calm space where participants were comfortable to share.

*“I feel these women are amazing and the way they can gently prompt us to grow and heal is so clever and it’s such a different approach. It sets the body up for long-term healing, it’s planting so many seeds and you take that with you for life”*

(Participant 5).

The facilitators’ caring and compassionate approach was described as:

*“…what you need when you are going through what we have gone through”*

(Participant 2).

### 3.4. “Came Together as a Perfect Puzzle”

#### 3.4.1. Practical Activities

Participants identified that the benefits associated with the practical components of *L2LA* helped them to heal from trauma and develop positive coping strategies. Practical components allowed women to *“release energy”* (Participant 2) and *“be able to let go and move on”* (Participant 3). Several participants specifically spoke about activities involving throwing a heavy rock and channelling negative thoughts/energy into that rock or mashing playdough.

*“We did a really good exercise where we had to pick up a rock and throw that and get rid of something we were holding onto; I suppose when you put in into something physical rather than thinking about it in your mind or doing something physically to get that out, I think it really helps”*

(Participant 3).

This was described as “*the best release I could have done*” (Participant 2). Women were encouraged to apply these strategies in their daily lives and share their experiences of using them.

Participants also discussed activities focused on speaking about their emotions and learning to regulate emotions as beneficial.

*“I really liked when we spoke about our emotions/feelings and how to control them.”*

(Participant 1)

Many activities also made use of tangible objects to help participants work through their emotions.

*“…those tangible things that we were given, were really helpful in visualising things in our lives”*

(Participant 1 Online).

Participants discussed various activities such as the empty cup and supportive jar aimed at reflecting on their current wellbeing and self-care practices, exploring how this impacts their capacity to give and support others. In the empty cup activity, participants filled cups with water to visually represent their level of fulfillment. Meanwhile, the supportive jar activity had participants identify their current support networks and place the details in a jar. Engagement in these activities was emotionally challenging activities for participants.

*“I already knew I was pouring from an empty cup, but until you see it with the water, you don’t, it wasn’t as obvious to me until them… it was a bit sad coming to terms that there wasn’t anyone to put in my jar”*

(Participant 1 Online).

#### 3.4.2. Self-Care

L2LA places a strong emphasis on self-care including what self-care is and looks like, which had a positive impact on the participants’ wellbeing.

*“Now, I wake up every morning and I try to give myself at least 10 min of meditation…it’s been super beneficial for my day not being so stressful… like I thought I was doing all the right things but after that course I could actually spoke to my friend and said I feel like I woke up, I’m actually me again”*

(Participant 2 Online).

Facilitators modelled self-care throughout the program creating a *“sanctuary”* with the use of oil diffusers, bean bags, cushions, and yoga mats. Participants in the face-to-face and online programs were also provided a care package. Participants reflected:

*“The ladies would always try to pamper us you know even just making us a cup of tea that was really nice like you felt really important”*

(Participant 2).

#### 3.4.3. Accessibility

Participants in the online and face-to-face programs valued the accessibility and consideration of their circumstances including childcare responsibilities.

The online program enabled the participation of women in other regional and rural areas of New South Wales. Participant 2 Online shared:

*“I wouldn’t have had access to the face-to-face version so I would have completely missed out on the whole thing, and it’s completely changed the way that I, it’s just really had a profound impact on my life”.*

The online version of the program facilitated access for women who feel uncomfortable in group situations. Participants appreciated the option to turn off cameras and leave calls if they felt uncomfortable. Participant 1 Online stated, *“I can just tap out if I need to”*.

Participants in the online program also discussed the benefit of not having to access childcare to attend.

*“I was able to have the kids in the other room whilst I was in the group, I didn’t have to stress about finding a babysitter for the kids, which would have been a difficulty an prevented me from engaging in the program if I was in a face-to-face group”*

(Participant 1 Online).

Participants in the face-to-face program were offered onsite childcare which facilitated their participation, “I wouldn’t be here if it was not for the group allowing the children to come” (Participant 5) and “it was beneficial having someone there to look after my child otherwise, I would not have been able to do it” (Participant 2).

However, one participant experienced challenges with onsite childcare arrangements stating,

*“I wish it was easier for me with my kids, I have been given every opportunity with the childminder here, but I think with my kids being so clingy and attached to my hip it can be a bit distracting as I am not able to fully focus”*

(Participant 5).

This participant felt that onsite childcare limited her ability to focus on the content and activities.

### 3.5. “Moving on and Moving Forward”

#### 3.5.1. Improving Wellbeing and Healing

Participants shared their motivations for engaging in the program which centred on improving wellbeing, healing, and moving forward for themselves and their children. These factors helped maintain their engagement and commitment throughout the program,

*“Given such an invaluable opportunity to be a part of something like this. I felt very lucky to be here and I saw it as a great opportunity to move forward for me and my kids”*

(Participant 5).

Similarly, Participant 3 engaged in the program to “better myself to move on and move forward for me and my daughter” (Participant 3).

Other participants felt that the program is unique and offered something different to other support services,

*“I feel there are not enough support services to access out in the community that are like this one”*

(Participant 6).

Participant 5 commented that other programs were, “like being in a classroom” and did not support their wellbeing and healing.

#### 3.5.2. Recommending L2LA

Participants acknowledged the positive impact L2LA had on their lives and recognized that it could also be beneficial for other women who have experienced domestic violence. *L2LA* was described as “*life*-*changing*” (Participant 4) and creating an “*opportunity to live a better life*” (Participant 5). Several participants elaborated that they would recommend the program to other women.

*“I would definitely recommend to any person experiencing domestic violence whether in their past, present, or future because I came out with such valuable skills and tools that I can use and very simple ones too that I can use going forward”*

(Participant 2).

Other participants discussed the desire to support other women to engage in the program stated:

*“I genuinely really want to make a difference and am because I’m kind of on the other side, I am a kind of a strong woman and the shit that I have gone through is really messed up… I’m safe now and I know I am completely safe now. So, for them to see me having a positive life as well”*

(Participant 2 Online).

### 3.6. Differences Between Online and Face-to-Face Programs

#### 3.6.1. Scheduling of L2LA

There was a significant difference in the length of the face-to-face and online program sessions. The face-to-face sessions were scheduled for 3 to 4 h for eight weeks during the school term. The online sessions were restricted to 30 min for five weeks during the school term. The majority of the participants in the face-to-face program were satisfied with the timeframe stating there is “*enough time to get through all the work and learn*” (Participant 1), and *“time to connect with other women”* (Participant 3). Another participant stated:

*“…at first I thought it was too long 3–4 h but I think I enjoyed it that much and I got that much out of it that I felt we could have gone longer”*

(Participant 6).

Participants in the online program felt that the time restriction limited the potential benefit of the program, particularly the capacity to share experiences.

*“Like this obviously had a really big impact on, but I think that there could’ve had been even a bit more time to share around our mutual sessions. So, at the end of session because each session only goes for half an hour”*

(Participant 2 Online).

#### 3.6.2. Concerns

There appeared to be more concerns for women engaging in the online program than the face-to-face program. The online program was initially delivered via a telephone conference before moving to Microsoft Teams and participants expressed that it limited their engagement and motivation to continue.

*“When you talk over the phone at the same time, like in person we can talk at the same time, and you know who’s talking and like with this you sort of have to hold yourself and some things are missed being said because you’re waiting”*

(Participant 2 Online).

### 3.7. Facilitators

Facilitators from both the online (*n* = 2) and face-to-face (*n* = 4) L2LA programs were interviewed to gather their insights on facilitation, highlighting what they felt worked well and the challenges. Two overarching themes were identified, (1) “Sitting in the space” which focused on creating safe spaces, supporting women to support their children, being flexible and enhancing wellbeing and (2) processes including the pre-group interview, post-referral process, scheduling of program, and safety planning. Please see Table 5, facilitator themes.

### 3.8. “Sitting in the Space”

#### 3.8.1. Creating Safe Spaces

Facilitators discussed the importance of creating a safe space for participants to enable women to sit in the L2LA space. Simply turning up to the program starts the healing process and empowers women to actively engage when they are ready.

*“We want them to turn up because even sitting within the space allows us to create safety and supports them… We don’t force them to do any activity because obviously, that’s the circumstance they’ve come from”*

(Facilitator 3).

Facilitators shared that creating a safe space also nurtured connections between women and facilitators.

*“It is more around hopes that they feel a sense of connection and the hope is that the facilities are able to create a safe space where there’s some space for healing and that at the end of it, the participants have some strategies that will support them.”*

(Facilitator 1).

Creating safety also supports women’s agency and helps to re-establish a sense of control over their lives.

*“…we want everything to be about their own choice and own power in the information and messengers that they are getting and receiving and giving as well…if they want to come, they can answer, they can talk when they want to, they don’t have to…”*

(Facilitator 1 Online).

Creating a safe environment in both face-to-face and online programs posed challenges, particularly in balancing the sharing of women’s experiences with the risk of triggering trauma.

*“… if someone starts talking about a recent incident of violence and we know that that’s going to be a massive trigger probably for the whole group, how we then allow her to tell her story, but then safely wrap it back up and reflect it on the issues is a challenge”*

(Facilitator 3).

Facilitators also shared that managing group dynamics could pose challenges to creating a safe space where all participants were able to share.

*“The most challenging components of facilitating is managing a range of trauma and managing the different dynamics of women. We often get a few that are big talkers and others are more introverted so finding the space for everyone to share if there are those powerful personalities is definitely one of the challenging parts of facilitating”*

(Facilitator 2).

#### 3.8.2. Supporting Women to Support Children

The L2LA program utilises the Safe and Together model enabling women to bring their children and have them cared for in a safe environment.

*“Lots of the groups in the Illawarra don’t offer childcare that runs alongside it. So, one of the things that we’ve tried really hard to do, even though we have no money is to use one of our other workers from our programs for childcare to be with the children in the adjoining space because it’s more that the women are likely to attend if they can be safe and together with their child”*

(Facilitator 3).

This model of care is unique in that the care of children is not viewed as childcare but as an opportunity to support children as well as their parents.

*“I would prefer it to be more of like that Theraplay style of stuff supporting the children rather than calling it childcare. So, when mum comes in and she’s really like anxious or having really overwhelming fear feelings. Then what we’re saying from the worker that’s sitting with the children is that the children are reflecting that.”*

(Facilitator 3).

Some facilitators also highlighted challenges with children attending particularly when the child preferred to stay with their parents.

*“… it was when kids would want to come in and out of the room… it was a challenge in terms of how that mum was then able to express herself, speak freely, and how the other women may have felt comfortable speaking with the children there”*

(Facilitator 4).

#### 3.8.3. Being Flexible

Facilitators discussed the flexibility of the *L2LA* program “*to meet where the women were at*” (Facilitator 3). Facilitators regularly adapted the program to meet women’s needs and fit the group dynamic.

*“What I think worked really well was how adaptable the program can be, so we had our plans or rough guideline of what each week was meant to be about, but it was very tailored to the group and where they were at on that particular day.”*

(Facilitator 4).

Facilitators noted that this flexibility made it well-suited for online delivery allowing women from other regions to access.

*“…it’s so easy to reach people in an online format, you know I can run this program anywhere, from anywhere to anyone anywhere, so I think it is an amazing way being able to deliver it”*

(Facilitator 2 Online).

The ability of facilitators to understand “*what is working for that group*” (Facilitator 2) and “*tailoring activities to the group*” (Facilitator 4) fostered women’s engagement and healing. For example, a facilitator reflected on a time when she adapted the ‘My kids and I—giving and receiving love activity which involved women writing a letter to their child.

*“…some women may not have their children in their care anymore and in week 7 we do a letter to your child so that might be quite triggering so we might not do that or adapt that”*

(Facilitator 2).

#### 3.8.4. Enhancing Wellbeing

The practical activities embedded in the L2LA program and tangible resources available to facilitators further enhanced women’s wellbeing. An activity that all facilitators shared as positive was a weighted therapy activity called Rock Week. One facilitator explained:

*“My favourite week is Rock Week. I like Rock Week because it’s one of the ones where they get to do lots of weighted activities. So, we use really heavy rocks and channel emotions and energy into these rocks and the women get to say whatever they want to these rocks, and they absolutely launch them outside”*

(Facilitator 3).

Facilitators found this activity very therapeutic for women as it gave them permission to express and release negative emotions.

Facilitators were provided with a toolbox including resources and tangible objects to use throughout the program.

*“… the toolbox is like a list or a tonne of little activities or experiences that you use throughout the eight weeks of the program, but it is not specifically scripted into a week”*

(Facilitator 3).

The use of these tangible objects and activities helped women to visually represent their emotions and understanding of emotions. For example:

*“…women were talking about how I don’t really know what love is and loves shit and loves this and loves that, so we go ok, the heart toolbox. So we go into our toolbox as facilitators and then we say to them, can you draw a heart and then we get them to on their page of their journal and we say put on the inside of your heart after so many weeks, what you think is actually love. And then on the outside, what you thought was love or what that person perceived to give you as love when it’s actually not”*

(Facilitator 3).

Facilitators of the online program posted personalised packages to each participant to use during the sessions. Similarly to the toolbox, these packages included tangible objects and resources to enhanced women’s wellbeing and facilitate the healing process,

*“…essential oil they could put on before they did the group, the little chocolate, the cup of tea or the coffee…which enriches the experience if it feels personalised for them and just you know, having it so easy to just grab what week you’re in”*

(Facilitator 2).

### 3.9. Processes

Facilitators identified processes within the program such as pre-group interviews, post-referral processes, and program scheduling that enabled women to participate safely.

#### 3.9.1. Pre-Group Interviews

Pre-group interviews help facilitators to understand women’s experiences, assess risks, and determine their suitability for the program. When necessary, safety plans were also implemented. Facilitators felt the pre-group interviews assisted with limiting risks or concerns for women.

*“… we do the pre-group interview so we understand their trauma and it makes us aware of potential triggers that there might be”*

(Facilitator 2).

Through the pre-group interviews, facilitators were also able to identify potential responses to participants if they became distressed.

*“We offer a space where they can fall out to if they need time out to process something and then one of the facilitators has done the pre-group interview, so we know whether or not that person needs to be followed to that safe space within that moment or if you give them 5–10 min and then go in and check”*

(Facilitator 3).

Facilitators emphasised that concerns can still arise despite having a pre-group interview process. One facilitator stated:

*“… that is what part of the pre-interview is to identify but there’s always an element of risk or things can change for them along the way”*

(Facilitator 1).

In addition to the pre-group interview, facilitators of the online program also called women before and after each session.

*“We are really checking in as we go along, we’re not leaving it straight away till the end, so that builds some rapport for the group even though it’s quite refined”*

(Facilitator 1 Online).

#### 3.9.2. Post-Referral Process

Facilitators discussed the post program referral process which links women to relevant support services to provide a continuum of care.

*“We do try really hard to make sure if they haven’t already got another support in place that they do that during the group, so that when it is over they are moving into something else, and we are not just kind of leaving them. Or we refer them onto one of our other groups like a parenting group”*

(Facilitator 4).

Facilitators recognised the importance of putting support in place for women to ensure that their support network is not lost once the *L2LA* program is over.

#### 3.9.3. Facilitator Debrief

Facilitators complete a debrief process with the co-facilitator after each *L2LA* session. Debriefing provided a space for facilitators to discuss what worked in the group and plan for the following week.

*“It was really important for the facilitators to debrief each week and have some space after the group. We definitely need to debrief and get together to plan for the next week given what had happened in that particular week. Having the time to do that is vital”*

(Facilitator 1).

The debriefing process allowed facilitators to collaboratively address any concerns or distress they experienced.

*“…if there is anything that came up for either of us, we discuss it in that space and if needs be we follow up with our manager. We are quite mindful of having that time after so you are not carrying their trauma with you, and you can offload together and move on”*

(Facilitator 2).

Debriefing supports facilitators in their role as it helps to mitigate trauma and burnout.

#### 3.9.4. Program Scheduling

Facilitators in the face-to-face and online programs expressed different views on the program’s scheduling. Face-to-face facilitators felt the program ran for an appropriate length of time and 2.5 h for each session was adequate,

*“Most women would like it to run longer than eight weeks and it is sad when it ends but eight weeks feels long enough, we get through a lot of material in that time and I don’t think we need it to be any longer and I don’t think it can be any shorter because some women need that amount of time to build confidence in the people around them and trust to share and begin to do that healing”*

(Facilitator 2).

The *L2LA* program is also strategically delivered during the school term from 10:30 to 1 pm to accommodates women’s childcare responsibilities.

*“We do it from 10:30-1 because it allows them to not be rushed in the morning and get to the venue on time. We also try to do it within a 10 or 11-week term but not starting week one of the term … so that if women have children in school or going to childcare that they can still get their kids to school”*

(Facilitator 3).

Facilitators of the online program commented that the scheduling of only thirty minutes per session over a five-week period was a significant limitation. Facilitators shared that each session often go overtime,

*“Thirty minutes for a group program is a really small amount of time, once you check in with everyone, once we you know allow them to have a discussion after the weekly activity occurred… it did not feel long enough. The women were pretty good to go over a lot of the time, which we needed, just to allow for them to have space to speak about their experiences”*

(Facilitator 2 Online).

#### 3.9.5. Safety Planning

Facilitators of the online program noted that there were safety concerns for the women, particularly about their perpetrators discovering their participation. The program was initially delivered as a teleconference via mobile phones and then moved to Microsoft Teams.

*“We didn’t know if the perpetrator would be within the home environment with them, when these calls were taking place… generally with the technology and the phone use, the calls can be deleted, taken off when they are checking their phone. But generally, if the perpetrator has access to their technology, they can usually see, whether it be their emails, or logging into Facebook if they doing a meeting that way…”*

(Facilitator 1 Online).

Facilitators attempted to mitigate the potential tracking of women through their devices by creating private groups.

Safety concerns were less prominent in the face-to-face program; however, facilitators noted that women’s circumstances can change, and they may no longer be suitable for the program.

*“I had to talk to her about that and how it was not ok for her to keep coming back to the group as it would have been quite damaging for the other women”*

(Facilitator 1).

Facilitators commented that the pre-group interview discussed previously usually identifies women who are still in contact with the perpetrator.

## 4. Discussion

The pilot study aimed to explore the experiences of participants and facilitators in the L2LA program, focusing on the experiences of women participating in and facilitating the program. The study identified several components of L2LA that provided positive experiences or created challenges. Overall, both participants and facilitators reported positive experiences with L2LA.

Consistent with the wider literature [40,41,42,43], the study found that L2LA had multiple benefits for participants. These included connecting with women in similar situations, learning tools and strategies to cope with their experiences, discussing their lived experiences of domestic violence, and receiving support in their recovery. Participants valued the opportunity to share their experiences and gain new perspectives from others, which aligns with findings from Brosi et al. [44] that intervention programs can help women change their perspectives on life. Participants also recommended L2LA to other survivors of domestic violence due to the benefits they experienced, reflecting their positive experiences with the program.

The L2LA program particularly enhanced social connections among women with children who had experienced domestic violence. Participants reported feeling less lonely and more validated, which contributed to their sense of worth, identity, and self-compassion [45,46]. This supports findings that group programs can reduce the stigma of mental health issues and provide a comfortable space for support [28,31,47].

Barriers to participation included work, time, and childcare, similar to those found in other studies [48]. Facilitators recognised the importance of offering childcare alongside L2LA to assist women in participating, as many other domestic violence programs in the Illawarra do not offer this. They also adjusted the program schedule to accommodate participants’ employment, which helped multiple participants attend the sessions.

The study suggested that programs providing supportive care through mother–child sessions can help women parent effectively [13]. Participants appreciated the program’s ability to indirectly assist with their children’s experiences of domestic violence and teach them therapeutic techniques learned from L2LA. However, facilitators noted that the childcare aspect could be more therapeutic, a finding supported by Jenney et al. [49], who found that many intervention programs provide childcare but lack therapeutic interventions between mother and child. Facilitators suggested that L2LA should consider offering mother–child sessions, supporting Hooker et al. [50].

Facilitators’ skills were crucial for effectively implementing activities and engaging participants in therapeutic activities. They used strategies such as flexibility, ongoing support, debriefing, and increasing program intensity. Kamal et al. [51] echoed these findings, suggesting that facilitators need skills to engage participants in practical components to help them cope with the aftermath of abuse. Consistent with the other literature [52], the findings suggest that facilitators should be selected based on their skills to ensure a successful intervention program. However, new facilitators faced challenges as they needed time to build their skills, reflecting Williamson and Abrahams’ [52] findings that program success depends on facilitator competence.

Safety planning was another crucial aspect of L2LA. Barnardos implemented a pre-group process to recruit suitable participants and plan for safety where necessary. Facilitators emphasized the importance of safety planning to reduce risks and ensure participants felt safe during and after the program. These results align with Jeffers et al. [53], who identified the importance of safety planning in domestic violence programs. L2LA employed similar safety planning strategies, such as conducting assessments, helping women identify safety threats, developing safety plans, linking women to support, and completing safety check-ins [54]. Despite these measures, challenges still arose due to group dynamics and participants sharing their experiences of domestic violence, similar to findings by Crespo et al. [40].

The study suggested that women who have experienced domestic violence are more likely to attend L2LA if they can be safe and together with their children [55]. Facilitators acknowledged the importance of L2LA being trauma-informed and understanding participants’ trauma, achieved through the pre-group process. Facilitators’ understanding and application of trauma-informed care, attachment, and grief and loss theories throughout the practical activities enabled them to work with women to reform and affirm attachments between mothers and children and acknowledge grief and loss as a process of reconstructing meaning in women’s lives [56]. This approach helped facilitators respond effectively to participants, providing a therapeutic, trauma-informed, and Safe and Together model that created positive experiences for participants [57].

The study explored participants’ experiences to understand their needs and what worked well within L2LA. Key aspects included connecting with other women in similar situations, practical components, learning coping skills, sharing experiences, and being in a safe and nurturing environment. Understanding these needs can assist with future implementation of the L2LA program [58].

Practical activities within the program, such as self-care diaries and meditation, enhanced engagement and helped participants incorporate these practices into their daily lives. This approach aligns with Hansen et al. [59], who suggested that psychoeducation can help women rebuild routines. The empowerment model used in the program encouraged women to reshape their lives and gain a sense of self through mindfulness and self-care [60]. Participants also expressed a desire to support others through their lived experiences, reflecting the theme of altruism discussed by Valpied et al. [61].

The study also discussed the benefits of engaging with the online L2LA program. Current studies [27,28,31] have highlighted the increased use of online intervention programs due to their accessibility. Participants found the online format beneficial, especially if they were restricted from attending face-to-face groups due to geographic location, childcare needs, or comfort in engaging from home. This accessibility is supported by findings from Ndungu et al. [27] and Milne-Ives et al. [62], who noted that online programs can reach more participants with fewer resources.

The online program also provided anonymity, which increased participants’ comfort and discretion [28,31,47]. Facilitators noted that using phone calls for the online program added a level of safety, reducing anxiety that might be heightened in face-to-face groups [63,64]. Participants appreciated the option to turn off cameras and leave calls if they felt uncomfortable or unsafe.

Despite these benefits, the study identified several barriers to engaging in the online L2LA program. The inability to see participants and facilitators via phone calls was a significant barrier to engagement and social connection. Participants found it challenging to know who was speaking, and distractions prevented full immersion in the program. This issue is supported by Strand et al. [64] and Irvine et al. [63], who noted difficulties in communication and reading body language in online programs. Facilitators also found it challenging to gauge participants’ emotional states without visual cues.

Another barrier was the limited time allocated for the online program, which participants felt was insufficient for discussions and free expression. This finding, although not widely supported by the existing literature, suggests a need for further research on time constraints in online programs.

### Strengths and Limitations

A notable strength of this pilot study was its contribution to understanding the experiences of both participants and facilitators in this unique program. It is essential to understand the small group nature of L2LA, its flexibility, and its quality, as well as its focus on empowering women to support their children. The pilot study captured diverse perspectives from participants and facilitators of the face-to-face and online versions of the L2LA program and highlighted what worked well and suggested areas for improvement for future iterations of the program.

However, the pilot study had limitations, notably the small sample size and low response rate. Twenty-four former participants opened the survey link; however, the response rate to each question varies considerably. Of the current L2LA program cohort only eight women participated in interviews and six facilitators. This small sample size reflects the program’s 4–6-person model which was purposeful to ensure a trauma-informed approach. The online version of the program only had a total of three participants. Recruitment was challenging as is often the case in hard-to-reach population groups [65]. We also considered contacting women, who left the program but were equally mindful this could put them at risk, future evaluations would need to consider how to include this subgroup.

These limitations overall restricted the generalisability of the study findings; however, despite these limitations we feel it was still critical to acknowledge that women’s voices were heard and relevant to future iterations of the program.

## 5. Conclusions

This pilot study is the first to explore the experiences and perspectives of women and facilitators in the L2LA program. It contributes new knowledge to domestic violence interventions aimed at supporting women and their children who have experienced DFV. The study identified several benefits for participants engaging with the face-to-face and online versions of L2LA program, including social connection with other women, self-care, development of coping strategies, re-connection with children and validation of experience. The online version enabled women in geographically isolated areas to receive support. However, there were some safety concerns, and the limited timeframe inhibited women’s ability to fully engage in the program. Facilitators highlighted several challenges, including managing trauma and the group dynamic within the sessions, while allowing women to share their experiences. The pre-group interview and de-briefing processes allowed facilitators to mitigate some of these issues and adapt the program when and as needed.

The L2LA program offers a supportive and empowering experience for women who have experienced DFV. In the future, the program may consider involving program components that involve children thereby enhancing family wellbeing. Future evaluations could expand upon this pilot study by thoroughly examining the differences between modalities. Additionally, future evaluations could aim to target a larger sample size and incorporate more rigorous outcome evaluation measures to comprehensively assess the program’s effectiveness.

## Figures and Tables

**Table 1:** Program topics.

Topic	Aim	Activities
Week 1: Introduction—what to expect	Gain an understanding of the program and how it supports participants	Group agreement Mindfulness activity (meditation) Islands of Safety activity; what does freedom from violence look like (use empty cup and supportive jar to visualise Islands of Safety) Introduce weekly journal
Week 2: Felt sense—turning into yourself	Explore personal strengths and help participants to begin to reconnect to their bodies	Strengths cards activity Islands of Safety: visited by women Mindfulness activity
Week 3: The Healing Tree—strength through growth	Builds on week 2 to help participants connect with their body to later help participants to learn to process trauma they have experienced	Strengths card activity Healing tree meditation Healing tree activity identifies individual’s supports, skills, beliefs/values, strengths, hopes, and dreams
Week 4: Letting go—the weight has lifted	Participants engage in activities to help them let go of negative energy	Strengths card activity Islands of Safety Release activity: release rock into a box/bucket Create individual or family identity shield
Week 5: Pathways of change—love myself	Participants identify their positive qualities and hopes and dreams for the future	Quality cards activity Islands of Safety Pathway of change activity: identifies supports, strategies and mechanisms for positive change and triggers for returning to perpetrator Love letter to yourself
Week 6: Patchwork life—goals and reality	Participants explore qualities that will support themselves and their children and identify goals	Breathing activity Island of Safety Cutting the cords and sharing wisdom Patchwork of Life Activity: create patchwork of identity diagram
Week 7: My kids & 1—giving and receiving love	Participants explore their strengths and their children’s strengths and the love that will help them to reconnect with their children	Strengths card activity: identify parenting strengths and children’s strengths Meditation activity Letter/card to children
Week 8: Reflection—pathways of change revisited	Participants reflect on their experiences of the program	Reflection and evaluation Review Pathway of change diagram
All weeks: Share morning tea with other participants, children and facilitators	Helps participants to develop sense of safety and trust between each other and with the facilitators	Journal time

**Table 2:** Study participants.

	Online Version of L2LA	Face-to-Face Version of L2LA	Total	Type of Data
Former participants	16	8	24	Survey
Current participants	2	6	8	Interview
Current facilitators	2	4	6	Interview
Total	20	18	38	

**Table 3:** Survey questions.

What motivated you to complete the program? Did you find this program beneficial? Do you feel the program’s delivery (including duration of sessions, the topic of sessions, and activities) was facilitated appropriately? Overall, how would you rate your experience taking part in the L2LA program? How engaged in the program did you feel? Did the program meet your expectations regarding what you hoped to get out of it? What topic did you find the least helpful? What topic did you find the most helpful?

**Table 4:** Participant themes.

	Themes	Description
1.	“Get some light on everything” (a) Making Connections (b) Providing a safe and nurturing environment (c) Facilitators’ connections with women	Focuses on the ways the program helps women build connections between each other, with facilitators, and to themselves by creating a safe and nurturing environment.
2.	“Came together as a perfect puzzle” (a) Practical Activities (b) Self-care (c) Accessibility	Focuses on how the program’s practical activities and focus on self-care enhance women’s healing and recovery. Making the program accessible by providing childcare and an online option is a piece of the puzzle missing from other programs.
3.	“Moving on and moving forward” (a) Improving wellbeing and healing (b) Recommending L2LA	Focuses on women’s accounts of how the program facilitates their healing and recovery, improves their wellbeing and their connection to their children allowing them to move forward and recommend the program to other women.
4.	Differences between the Online and Face-to-Face Facilitation (a) Scheduling of program (b) Concerns	Outlines the main differences between the face-to-face and online programs identified by women.

**Table 5:** Facilitator themes.

Theme	Description
Sitting in the space	The focus is on how the program creates safe spaces, supporting women to support their children, being flexible and enhancing wellbeing.
2. Processes	Focuses on the practical processes the facilitators identified as important to the program’s success including the pre-group interview, post-referral process, the scheduling of the program, and safety planning.

## Data Availability

The data presented in this study are available on request from the corresponding author.
